# A large subchorionic hematoma in pregnancy: A case report

**DOI:** 10.1097/MD.0000000000020280

**Published:** 2020-05-29

**Authors:** Yu Liu, An Tong, Xiaorong Qi

**Affiliations:** a Department of Gynecology and Obstetrics, Development and Related Diseases of Women and Children Key Laboratory of Sichuan Province, Key Laboratory of Birth Defects and Related Diseases of Women and Children, Ministry of Education, West China Second Hospital; b West China School of Medicine, Sichuan University, Chengdu, PR China.

**Keywords:** intrauterine mass, pregnancy outcome, subchorionic hematoma, ultrasonography

## Abstract

**Introduction:**

Subchorionic hematoma (SCH) is a rare type of intrauterine hematoma, usually with limited impact on fetuses and pregnant women. But massive hematoma causes significant space occupying effect, affects blood supply of the fetus and finally may lead to fetus demise.

**Patient concerns:**

In this case report, we reported a 22-year-old pregnant woman presented to our hospital with complaint of irregular lower abdominal pain.

**Diagnosis:**

Ultrasonography and magnetic resonance imaging confirmed an intrauterine mass with a compressed growth-restricted fetus.

**Interventions:**

The patient underwent diseases induced labor after confirmation of fetus demise by ultrasonography.

**Outcomes:**

Histopathological examination of the mass revealed a SCH.

**Conclusion:**

Though small SCH can be found in quite a few pregnant women and is usually harmless, enormous hematoma can result in adverse pregnancy outcomes. It may be difficult, in some cases, to differentiate it from uterine tumors or placental tumors by means of ultrasonography and magnetic resonance imaging, especially when the mass is hyperechoic under ultrasonography. This case report stresses the importance of regular examinations of pregnant women.

## 1 Introduction

Intrauterine hematoma (IUH) is an pregnancy event resulted from thrombosis, anticoagulant therapy and more often, unknown reasons.^[1]^ IUH can be further divided into subchorionic hematoma (SCH), retroplacental hematoma and subamniotic hematoma according to its location, among which SCH is relatively more common than the other 2.^[1]^ Small hematoma may be asymptomatic and can be discovered during routine obstetric ultrasonography,^[2]^ but larger ones can be characterized by abdominal pain, vaginal bleeding or discharge, decreased fetal movement and other atypical symptoms.^[3]^ Clinical significance of such hematoma is controversial, some adopting the view that it exerts limited influence on the ongoing pregnancy, while others holding concerns about risks of increased complications.^[4–6]^ What is consistently agreed is that small SCH needs to be followed up by ultrasonography in case of its enlargement.^[7–9]^ But another more hazardous form of SCH, Breus’ mole, characterized by massive hematoma more than 1 cm thick, is undoubtedly to trigger adverse pregnancy outcomes, such as intrauterine growth retardation, fetal demise, placental abruption, premature rupture of membranes.^[3,10,11]^

## 2 Case presentation

A 22-year-old pregnant woman presented to this hospital at 20 weeks of gestation (gravida 1, para 0) with irregular lower abdominal pain. The patient had been at her usual menstrual cycle till about 20 weeks before this admission, and she was not sure about the accurate period of amenorrhea. She had not paid routine prenatal visit to the obstetrics. About 10 days before this presentation, the patient complained of radiating lower abdominal pain with no obvious predisposing cause, and there was no vaginal bleeding or discharge. It was not until one day before this admission that she went to the local hospital for medical help. Ultrasonography in that hospital confirmed her pregnancy and simultaneously found a 17.8cm × 10.6cm × 15.4 cm solid mass in her uterus. Once the mass was found, the possibility of uterine leiomyoma or even malignant tumor was taken into consideration. So the patient was transferred to obstetrics department of this hospital for further evaluation.

**Figure 1:**
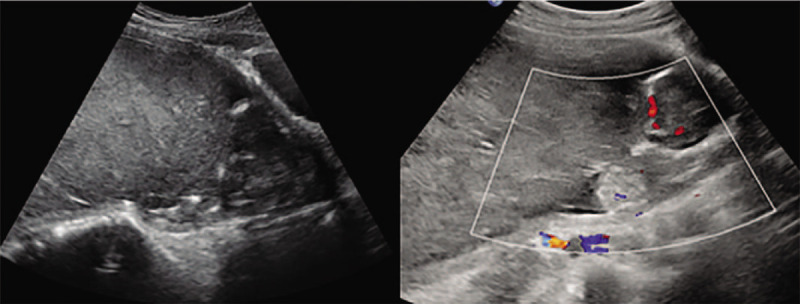
The sonogram showed a huge hyperechoic mass occupying majority of the uterine cavity and the fetus was pushed to the internal orifice of cervix.

On the patient's arrival at this hospital, limited history could be obtained due to language barrier. There was no report of nausea, vomiting, edema, vaginal bleeding or discharge. She took no medications and had no known contact history to toxicant. There was no family history of tumor and teratism. On admission, her vital signs were stable. Irregular uterine contractions was palpable on her lower abdomen and pelvic examination showed anterior cervical position, 80% effacement and no dilatation of cervix. Laboratory test results showed hemoglobin 114 g/L, red blood cell count 3.8∗10^12^/L, white blood cell count 5.8 × 10^9^/L, neutrophil percentage 59.6%, platelet count 244 × 10^9^/L, fibrinogen 259 mg/dL, D-dimer 404 μg/L and total human chorionic gonadotropin (HCG) 30637.2mIU/mL. Ultrasonography showed the live fetus with a crown-rump length of 7.44 cm, a biparietal diameter of 3.1 cm and a femoral length of 1.25 cm, but without normal placenta. Additionally, a solid hyperechoic mass measured 18.1 × 8.9 × 15.7 cm in the amniotic cavity was showed under ultrasonography, oppressing the uterus wall (Fig. 1).

We discussed the probable diagnosis of this patient. Considering the detected solid mass which oppressed the uterus wall, the possibility of tumor first came into our mind. Besides, a special circumstance called red degeneration of leiomyoma, mostly happening in gestation, was another candidate diagnosis. When conditions like a history of menopause, an elevated hCG, the missing placenta and the huge solid mass were all taken together, lesion originated from placenta became the criminal suspect. We especially talked about the possible diagnosis of partial hydatidiform mole, but it was disproved by an hCG lower than 100,000mIU/mL and a lack of associated ultrasound findings such as snowstorm pattern. On this occasion, we need to be cautious about establishing the diagnosis of partial hydatidiform mole. Curettage would be appropriate if it was exactly a partial hydatidiform mole, otherwise hysterectomy would be inevitable if it was a malignant tumor.

**Figure 2:**
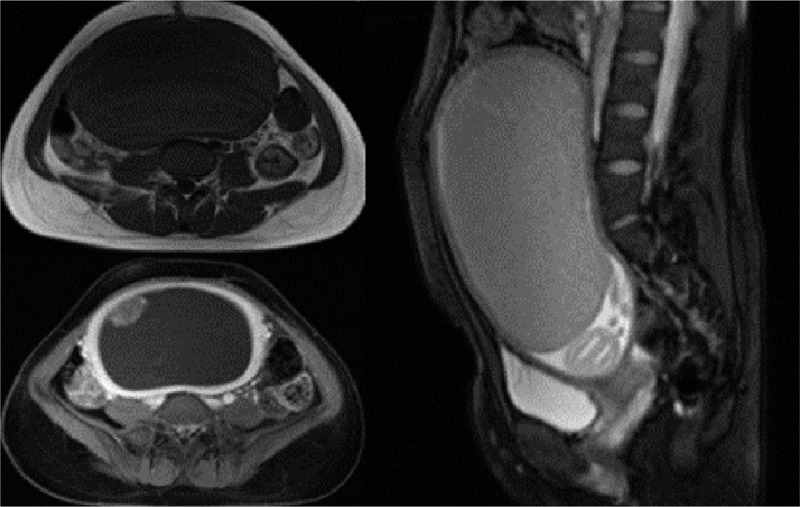
The pelvic MRI showed a 17.6cm × 8.4cm × 16.9 cm mass with long T1 and slightly long T2 signals in the uterus, of which only a small part was enhanced under contrast, and the boundary around the mass separated it clearly from the uterus wall. Still could the fetus be found at the internalorifice of cervix. MRI = magnetic resonance imaging.

To verify our conjecture and prevent misdiagnosis, another ultrasonography was conducted 3 days later. An extra tube-like echoless area with blood flow signals was detected. In the meantime, the patient complained of exacerbation of abdominal pain. Her hemoglobin decreased from 114 g/L to 97 g/L, fairly low in terms of people living in plateau, so 2 units of suspended red blood cells were transfused. Afterwards, the patient and her family decided to give up gestation. Therefore, enhanced magnetic resonance imaging was planned to figure out whether there was any invasion of the intrauterine mass into myometrium and to evaluate its association with surrounding tissues. The placenta was still missing and a 17.6cm × 8.4cm × 16.9 cm cystic mass with smooth border and homogenously enhanced thick wall presented in the uterus. The anterosuperior part of the mass was composed of cotton-shaped solid portion, enhancing significantly. No signs of invasion into myometrium was shown (Fig. 2). Based on these findings, lesions originated from placenta was also considered by radiologist. Besides, the possibility of twin reversed arterial perfusion sequence could not be excluded.

On the fifth day, patient condition deteriorated, her blood pressure dropped and her hemoglobin fell to 90 g/L. Bedside ultrasonography was performed, showing that the mass was inside amniotic cavity which appeared as large sheets of hyperehoic area with tube-like echoless area containing blood flow signals. The main difference was some space separating the mass from uterine wall. At this point, the possibility of hemorrhage was considered. Only after a detailed inquiry of her medical history did we get to know that one day before the onset of pain, the patient had carried stones which compressed her abdomen. So the diagnosis of IUH was extremely likely. Unfortunately, the fetus was confirmed dead under ultrasonography.

**Figure 3:**
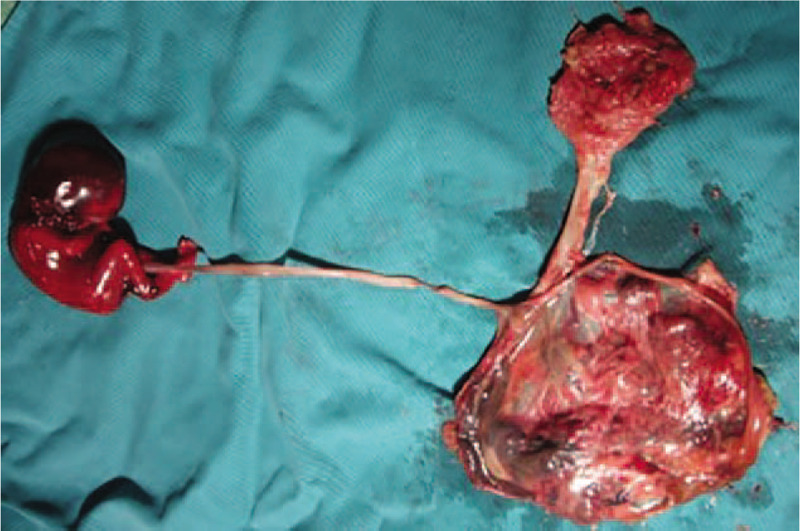
The dead fetus was delivered with a bilobate placenta (a main lobe and an accessory lobe) and a hematoma in the main lobe could be easily seen.

The patient's condition taken into consideration, induced labor was the only option. Therefore, COOK cervical ripening balloon was adopted to dilate the cervix and facilitate labor induction. One day later, the fetus along with duplex placenta was delivered and massive blood clots were found on the maternal surface of placenta at the main lobe (Fig. 3), verifying our conjecture. Ultrasonography confirmed that there was no residual fetal tissues in the uterus and her vital signs were stable, so the patient was sent back to the ward from the labor room.

Gross examination of placenta showed a main lobe of placenta duplex measured 12cm × 10cm × 1 cm and an accessory lobe measured 6cm × 5cm ×1 cm. Tissues of the main lobe was incomplete and only a few placental lobules could be seen. A 4cm × 3 cm hemorrhagic area was found under fetal membrane of main lobe, which isolated fetal membrane from placental lobules. Tissues of the accessory lobe were complete with focal infarction. Histopathological excluded the possibility of tumor but confirmed the diagnosis of SCH with severe chronic chorioamnionitis.

A month later, the patient received another determination of serum hCG which turned to be normal and her uterus acquired normal involution. The patient was pregnant and gave birth to a healthy child 2 years later.

## 3 Discussion

According to Williams Obstetrics, SCH is the synonymous to subchorial thrombus, which should be differentiated from subchorionic hemorrhage namely and retroplacental hematoma in location. Subchorionic hemorrhage, or more precisely marginal hematoma, is referred to those hematomas between the chorion and decidua at the placental periphery and used only clinically, such nomenclature really making things confusing. On the other hand, retroplacental hematoma occupies the space between placenta and its adjacent decidua, mostly caused by hemorrhage of small vessels in basal plate of the maternal floor. Additionally, a more infrequent type of intrauterine hematoma called subamnionic hematoma should also be differentiated from SCH in location, which deposits between amniotic membrane and chorionic membrane.^[12]^ To be consistent with the widespread definition of SCH, here we use SCH to refer to hematomas along the roof of the intervillous space and beneath the chorionic plate.^[8,12]^

Ultrasonographical findings of hypoechoic area in the placenta with space occupying effect are enough to establish the diagnosis of SCH. For massive hematoma, patients may have changes in red blood cell counting, coagulation function and symptoms like lower abdominal pain,^[13]^ but these are common in massive intrauterine hematomas. In some cases with massive hematomas, even ultrasonographical findings can not differentiate SCH from retroplacental hematoma, so we still have to rely on histopathological examinations.^[11,14–16]^ But it is unnecessary and valueless to accurately find the initial bleeding part in those cases with massive hematomas, because massive hematomas always resulted in poor outcomes of the fetus irrespective of the source of bleed.^[8,14–16]^

SCH usually occurs in the first trimester and its incidence ranges from 0.46% to 39.5% during natural conception,^[17,18]^ while patients undergoing in vitro fertilization (IVF) usually suffer from higher risk of SCH with an incidence ranging from 4% to 48%.^[19]^ Apart from those attributed to thrombosis and anticoagulant therapy, most SCH occurs spontaneously and no certain cause can be distinguished. It is postulated that a poorly developed vascular bed characterized by shallow trophoblast invasion and friable blood vessels, during implantation and angiogenesis of placenta, is to blame.^[8]^ It is the likely reason that can explain the higher incidence after IVF than natural conception. Furthermore, it has been retrospectively studied that compared with froze-thawed embryo transfer after IVF, immediate embryo transfer resulted in higher prevalence of SCH.^[20]^ Since immediate embryo transfer usually means poorer receptivity of endometrium and maternity than froze-thawed embryo transfer, it contributes to insufficient implantation and placentation, this outcome further evidencing the mentioned hypothesis. Another study also found that predisposing factors for IUH included uterine malformations, myomas, history of recurrent pregnancy loss and infections.^[21]^

Small SCH is usually discovered by ultrasonography, while massive SCH may have symptoms like abdominal pain, vaginal bleeding and decreased fetal movement. When the diagnosis of SCH is established, it is difficult to tell whether the hematoma will be absorbed spontaneously or persistently exist as well as whether it leads to adverse pregnancy outcome. Some studies demonstrated that compared with retroplacental hematoma, SCH had relatively better prognosis.^[22,23]^ The prognosis of SCH has been studied by several cohort and case-control studies. It showed that SCH was associated with increased risk of abortion, stillbirth, abruption, preterm birth and preterm premature rupture of membranes.^[8]^ Exceptionally, a study carried out in IVF patients found that SCH brought about lower birth weight in singleton pregnancy but did not increase pregnancy loss rate.^[20]^ As for prognostic value of variable factors, different studies acquired different outcomes. A retrospective study included IUH in the second and third trimesters found that maternal age, gestational age at first diagnosis, location of hematoma and accompanying contraction rather than hematoma volume or maternal parity were risk factors for poor prognosis.^[10]^ However another study figured out that subjective hematoma size based on the fraction of gestational sac size and gestational age at first diagnosis were the most predictive index of poor pregnancy outcome.^[24]^ There was also a study which found that the hematoma location relative to the placenta as well as uterus and duration of SCH had strong predictive value on the prognosis of the ongoing pregnancy.^[3]^ Different from those common SCH, Breus’ mole always resulted in adverse pregnancy outcomes.^[11]^

For most SCH patients, a strategy of follow-up and observation is appropriate, and bed-rest may provide possible benefit.^[7]^ Although vaginal lipoic acid and progesterone have been used to treat SCH and symptoms of threatened miscarriage,^[25,26]^ such treatment modalities still lacking of strong clinical evidence.

There are several causes of gestational occupying lesions in uterus, including gestational trophoblastic diseases, SCH, twin reversed arterial perfusion sequence, placental sinus, placental teratoma and so on. In this intriguing case, due to the lack of preconception examination results, we are unable to make sure whether the mass had existed before conception and whether it was related to pregnancy. Neither could we estimate the growth rate of the mass. These all brought difficulties to diagnosis. When we thought about the possibility of a pre-existing tumor before pregnancy, there was no supporting symptoms of vaginal bleeding or discharge, but without preconception examinations we still could not rule it out. Therefore, we considered the possibility of leiomyoma, especially red degeneration of leiomyoma. But the patient had no history of menometrorrhagia before pregnancy, no severe abdominal pain and no elevation of white blood cell count during gestation, which was not consistent with manifestation of red degeneration. Next, placental teratoma can also be excluded due to lack of the typical characteristic of calcification. As for twin reversed arterial perfusion sequence, it refers to pregnancy of monochorionic twin, where one of the twin is acardius (recipient) and the other (donor) pumps blood to it. A prerequisite of vascular anastomoses connecting their vascular systems makes it possible. However, in such cases, their placenta is usually detectable and normal in appearance, which prompted us to consider other possible diagnoses.

It seems that possibility lies on gestational trophoblastic tumor and hematoma. At our first glance of this case, the mass was huge and hyperechoic, in the absence of placenta. Since hematoma are hypoechoic, we postulated that the mass could be a placental tumor. On the other hand, an hCG lower than 100,000mIU/mL and lack of typical ultrasound findings such as snowstorm pattern still left the diagnosis of partial hydatidiform mole questionable. While the final result proved its hematoma identity, we speculate that the hyperechoic feature of this hematoma may result from hyperhemoglobinemia and hypercoagulability which is characteristic of people living at high altitude.^[27–30]^ Placenta was compressed by the huge hematoma and appeared as hyperechoic mass together with hematoma under ultrasonography, which caused the disappearance of placenta. Such huge SCH, along with those aforementioned misleading conditions, indeed, posed tremendous challenge to diagnosis.

In retrospect, ultrasonography parameters like crown-rump length, biparietal diameter an femoral length indicated an intrauterine growth retardation. Decrease of hemoglobin evidenced bleeding. Finally, the poor placental function of this patient could not support the subsistence expense of the fetus, and the bad outcome was inevitable.

## Author contributions

**Conceptualization:** Xiaorong Qi.

**Writing – original draft:** Yu Liu, An Tong.

**Writing – review & editing:** Yu Liu, An Tong, Xiaorong Qi.
